# The Treatment of Chronic Varicose Ulcers of the Leg

**Published:** 1896-09-26

**Authors:** W. McAdam Eccles

**Affiliations:** Assistant Surgeon to the West London Hospital, and City of London Truss Society, Surgeon to the St. Marylebone General Dispensary, and Demonstrator of Operative Surgery at St. Bartholomew's Hospital


### Medical Progress and Hospital Clinics.

[ *The Editor will be glad to receive offers of co-operation and contributions from members of the profession. All letters should be addressed to* The Editor, at the Office, 428, Strand, London, W.C.]

### THE TREATMENT OF CHRONIC VARICOSE ULCERS OF THE LEG.

By W. McAdam Eccles, M.S., F.R.C.S., Assistant Surgeon to the West London Hospital, and City of London Truss Society, Surgeon to the St. Marylebone General Dispensary, and Demonstrator of Operative Surgery at St. Bartholomew's Hospital.

Some of the common and frequently occurring ailments of the body are those which have defied successful treatment at the hands of many practitioners. This is most undoubtedly true of the affection known as the chronic ulcer of the leg due to varicose veins of the lower extremity.

While varicosity is prone to be present in persons of all ranks and of both sexes, yet it is particularly the working classes who are frequently attacked by the distressing condition brought about by an ulcer.

The dilatation of the superficial veins leads to much failure in the proper nutrition of the lower limb, and this particularly so near the ankle. As a result a crop of chronic eczema associated with much pigmentation often follows. I am inclined to think that the impeded return of the blood supply to the skin frequently sets up irritation, which leads the patient to scratch the part, and that the eczema is the outcome of this manipulation. Be that as it may, it is a certain fact that a person suffering from varicose eczema, as it is termed, is in a state which may be called a pre-ulceration one. A slight excoriation produced by rather more energetic scratching of the irritable part, or a blow causing a slight breach of surface, is, as it were, a match to the fire, the starting point of a trouble which may last for years, and be so resistant of cure that it becomes a heavy burden to the sufferer, and may lead him to despair as to the possibility of complete relief short of losing the offending part.

An able-bodied man, the bread-winner of the family, is thus laid aside from work, or is only able to perform his duties in chronic misery; a woman endures untold suffering for months, even years, is often under treatment, and gets no better.

Such are the oft-told tales of woe, all the outcome of a chronic ulcer of the leg. And yet one has to confess that these cases are very trying ones for the medical man to treat, and equally disappointing in the poor success which so persistently attends all his efforts.

What is at the bottom of all this failure? Some say it is want of rest to the part; others state it is lack of oxygen, and urge that a supply of this gas will rapidly bring about the desired healing; and again, others look upon the varicose veins themselves as the real factor in causing the continuance of the trouble. No doubt that possibly all these play a part, but one is forced to the conclusion that the septic condition of the ulcer is far and away the most important reason for its failure to cicatrise, and the very extension even of the sore. It is an every-day observation that a septic ulcer is unhealthy to the utmost degree; it discharges copiously, it is very painful, it is surrounded by a wide area of acute inflammation, or of chronic induration, and instead of showing signs of diminishing in size, it steadily but perceptibly increases in area. If therefore the patient is to be efficiently treated it must be our aim to bring about asepsis, or at least to lessen the amount of septic material present as soon as possible.

A patient presents himself with a varicose ulcer; he is told to keep it clean, to apply some ointment daily, and to get as much rest as possible. He follows the directions given to the best of his ability—though this may undoubtedly be small—and he finds little or no relief, and returns much dissatisfied, and to instil the same feeling into his medical attendant.

I am convinced that a careful and thorough treatment of such a patient by the surgeon himself, with a view to the extermination of the septic organisms, would lead to a large number of the beds in our infirmaries and hospitals being emptied of their occupants.

How then are these cases to be dealt with? I would venture to detail the line of treatment, more or less on the lines of Professor Unna's method, which I have found to be eminently satisfactory.

It must be clearly understood that this way of treating ulcers of the leg requires personal attention on the part of the practitioner himself, takes up a considerable amount of time, and needs somewhat expensive materials.

Briefly, the following are needed: Soap and water, carbolic acid solution 1-40, or, better, perchloride of mercury solution 1-2,000, carbolic gauze bandages, a modified Unna's paint or paste, web bandages, an ordinary glue pot, and a good sized paint brush.

The paint or paste used consists of boracic acid, zinc oxide, gelatine, glycerine, and water.

The following are the proportions of these several ingredients and the method of making the paste: ℞—Pulv. acidi borici, 1 part; pulv. zinci oxidi, 5 parts; glycerini, 8 parts; gelatini, 5 parts; aquæ distillatæ, 6 parts—all measured by weight. Rub down in a mortar the zinc oxide and boracic acid with a portion of the water and glycerine. Dissolve the gelatine in a saucepan with the remainder of the water and glycerine by the aid of heat. When dissolved mix the two together, and while hot pour into a suitable mould, such as a shallow metal dish. When cold it will be found to have set into a firm jelly, and may be cut into pieces of a convenient size. The process of dealing with the ulcerated leg must now be considered. The whole surface from the foot to the knee should be thoroughly scrubbed with soap and water, a cleansing which has probably not been its lot for many a day.

This will remove ordinary dirt and the accumulations of dried discharge, decomposing ointment, and other *debris* around and upon the ulcerated surface. This process of cleansing will give rise to some pain when the actual sore is washed over, but this is essential to ultimate success, and must therefore be endured by the patient.

The ulcer and a wide area around it is next to be swabbed with one or other antiseptic solution. Even at this stage of the proceedings the ulcer will seem to look more healthy, and it is certainly much cleaner.

Then carbolic gauze bandages are applied, including the foot and passing right up to the knee. There should be at least four layers of gauze over the ulcer itself, and two layers elsewhere.

The paste has, during these manipulations, been melted in the glue-pot, and is now ready to be applied hot; care, however, being taken that it is not of such a temperature as to scald the leg.

It is to be applied from the ankle to well above the site of the ulcer, and round the complete circumference of the leg. No very thick layer is needed; in fact, it is an objection to have too liberal a quantity laid on. Another layer of gauze bandage should be placed over the paste, and outside all, from the foot to the knee, a web bandage is smoothly adjusted with a fair degree of tightness.

As the paste cools it contracts, and therefore the limb is encased in an antiseptic dressing, producing a beneficial degree of pressure on and support of the veins and the ulcers.

The patients are directed that they must in no way interfere with the dressing, and that they may follow their ordinary employment. After three days they should be seen again by the surgeon, especially if, from the ulcer being very septic, there is likely to be a large quantity of discharge.

By means of a pair of ordinary dressing scissors the whole thickness of the dressing is cut through from above downwards on the side away from the ulcer, and thus removed. The surface of the ulcer will probably even by this time be covered with healthy granulations, and around the margins epithelium is developing. Another thorough cleansing should be undertaken, and the limb dressed in precisely the same way as before.

It may then be left untouched for a week or more, because the less septic the ulcer becomes the less discharge is given off, and the fewer times the dressing needs to be changed. The healing is astonishingly rapid, and the most unpromising cases often yield the best results. From a considerable experience I am now prompted to put every chronic ulcer of the leg, however long standing, or of however wide extent, under this treatment, and I have but rarely found it fail to considerably improve, if not completely cure, all the cases; even an ulcer which encircles the entire circumference of the limb is not hopeless.

